# Finding the best hardware configuration for 2D SLAM in indoor environments via simulation based on Google Cartographer

**DOI:** 10.1038/s41598-022-22938-y

**Published:** 2022-11-05

**Authors:** Łukasz Sobczak, Katarzyna Filus, Joanna Domańska, Adam Domański

**Affiliations:** 1 Simulation Departement, OBRUM Sp. z o.o., 44-117 Gliwice, Poland; 2 Institute of Theoretical and Applied Informatics, Polish Academy of Sciences, 44-100 Gliwice, Poland; 3 Department of Distributed Systems and Informatic Devices, Faculty of Automatic Control, Electronics and Computer Science, Silesian University of Technology, 44-100 Gliwice, Poland

**Keywords:** Computer science, Engineering

## Abstract

One of the most challenging topics in robotics is simultaneous localization and mapping (SLAM) in the indoor environments. Due to the fact that Global Navigation Satellite Systems cannot be successfully used in such environments, different data sources are used for this purpose, among others light detection and ranging (LiDARs ), which have advanced from numerous other technologies. Other embedded sensors can be used along with LiDARs to improve SLAM accuracy, e.g. the ones available in the Inertial Measurement Units and wheel odometry sensors. Evaluation of different SLAM algorithms and possible hardware configurations in real environments is time consuming and expensive. In our study, we evaluate the accuracy of mapping and localization (based on Absolute Trajectory Error and Relative Pose Error). Our use case is a robot used for room decontamination. The results for a small room show that for our robot the best hardware configuration consists of three LiDARs 2D, IMU and wheel odometry sensors. On the other hand, for long hallways, a configuration with one LiDAR 3D sensor and IMU works better and more stable. We also described a general approach together with tools and procedures that can be used to find the best sensor setup in simulation.

## Introduction

One of the most challenging topics in Robotics and Computer Vision is to enable autonomous robots and vehicles to navigate in unknown complex environments. To make it possible it is necessary to build a map of such environments and simultaneously determine robot’s or vehicle’s location within the created map. This procedure is called Simultaneous Localization and Mapping (SLAM), which is one of the core tasks of an autonomous robot or vehicle, as many different applications strongly depend on the generated maps^1^.

Autonomous systems operation is possible due to data gathered from multiple embedded sensors, mainly, LiDARs (Light Detection And Ranging), radars, cameras, odometry sensors, IMUs (Inertial Measurement Unit), and GNSS (Global Navigation Satellite System). Information gathered using these sensors is utilized in the four main components of autonomous systems (localization and mapping, understanding of the surrounding environment, path determination and vehicle control).

Safety of autonomous vehicles is crucial, and to achieve it, exact position and orientation are necessary, thus localization is one of the most important aspects of the autonomous systems. For localization, GNSS is often used, however it requires a GNSS receiver with an unobstructed line of sight to minimum four GNSS satellites to work properly^2^. Even high-end GNSS-based systems suffer from multi-path interferences (as a result localization of the vehicle can jump up to a few meters)^2^. In the domain of Simultaneous Localization and Mapping, indoor environments are a major challenge, because GNSS cannot be used to obtain an absolute position due to Radio Frequency signal blocking. Therefore, other technologies are used as a basis of the SLAM system for indoor environments.

In practical applications, LiDARs have advanced from numerous other sensors and technologies that were not sufficiently sensitive and accurate^3^. LiDARs determine distance to other objects by emitting laser beams and measuring their travelling time. Based on these readings, clouds of points are created that represent the surrounding environment. LiDARs offer wider field of view, as well as higher resolution than radars and ultrasonic sensors. Their operation is also more robust under different conditions, including light and dark environments, with and without glare and shadows^4^. This is crucial for vehicles and robots working in demanding environments, e.g. underground ones. Also, the closer spacing of light beams results in LiDARs having better angular and temporal resolutions than Radars^3^. In opposite to camera-based sensors, they offer more robustness and less noisy data. Also, they are less sensitive to changes in the lighting. All of these features make LiDARs the most reliable data source for the SLAM algorithm input^5^.

SLAM approaches can also be based on a fusion of data from LiDARs and different sensors, e.g. IMUs (Inertial Measurement Unit) and odometry sensors. These approaches aim to improve SLAM accuracy by estimating their position relative to a starting point. IMUs are often used in SLAM algorithms to provide an initial pose estimation of the robot (this initial pose is used for the scan matching problem), which significantly improves accuracy and stability of data association in dynamic environments^6^. IMUs, in contrast to GNSS systems do not rely on external information sources (which can be blocked or disturbed) and can provide information such as velocity and position based on the accelerometer and gyroscope readings over time. Nevertheless, inertial sensors suffer from drifts, therefore localization systems based on IMU data are subject to a rapid degradation of position over time^2^.

Also, data from other on-board sensors can be used to estimate changes in orientation and position relative to an initial location of the robot—e.g. wheel odometry sensors. In the case of wheeled vehicles, odometry is based on the changes in the movement of wheels. Here, rotation optical encoders or the ones based on Hall effect can be used and knowing the diameter of a wheel, its approximate linear displacement can be calculated. Based on wheels’ translation and the distance between the wheels, pose of the robot can be obtained. Also, the rotation angle of each wheel is calculated based on the encoder data in real time. Unfortunately, wheel odometry usually suffers from errors due to the integration over time resulting in the final pose estimation accuracy being poor and noisy, thus other sensors have to be used to obtain an accurate localization system^7^.

Aside from input data, many different approaches have been proposed to solve the Localization and Mapping problem. These approaches are usually most of which can be categorized into the following two methods: filtering-based and optimization-based ones^1^. The examples of filtering-based approaches are Extended Kalman filter or particle filters. They are used due to the fact that sensor data suffers from inconsistency and noise and these approaches allow modeling of different noisy sources and how they affect the measurements. The second group of methods—optimization-based one gained much popularity due to their effectiveness, robustness, scalability and better stability that the one of filtering-based approaches^1^. In such approaches measurements are usually represented in a form of a graph, which nodes represent poses of a robot and edges represent spatial constraints between different poses^1^.

One of the optimization-based approaches is Google Cartographer. It is one of the leading SLAM algorithms, which is compatible with Robotic Operating System (ROS)—a commonly used system in the field of robotics^8^. Cartographer can operate based on 2-dimensional and 3-dimensional LiDAR point clouds. The system is based on two types of SLAM: local and global SLAM. Local SLAM is responsible for matching the scans, incorporating adjustments regarding motion (angular and linear), creation of sub-maps and determining current trajectory^9^. Global SLAM, on the other hand, executes sparse pose adjustment, matches these created sub-maps and then produces a global map^10^. It is also used to detect loop closure—a common issue in the autonomous driving area—and to correct the global map in the case of detection^11^. As a result of the algorithm, the coordinates of the vehicle and the surrounding map are produced and sent to a navigation algorithm.

In this work, we present the cost-efficient evaluation methodology that can be used to test and compare different SLAM algorithms based on data from LiDARs, IMU and odometry. For that purpose, we utilize a simulated environment introduced in^12^. The framework was comprehensively evaluated in that study. For that purpose, we compared the measurements obtained with simulated and actual real-world devices in real-life scenes. It was proven that the proposed framework delivers very accurate and realistic data, and also that the results obtained via simulation can be reproduced in the real world. In this article, we use this previously verified simulator to compare different hardware configurations of Google Cartographer SLAM algorithm (we use different subsets of the following devices: LiDARs 2D and 3D, IMU and wheel odometry). We chose this particular SLAM algorithm due to the fact that it can operate using various data sources (2D and 3D LiDAR, IMU, odometry). Also, in many research papers Google Cartographer obtained the best results out of all examined algorithms. e.g.^13,14^. We compare the performance of this SLAM algorithm operating with different hardware configurations based on the accuracy of generated maps, localization errors (we used Absolute Trajectory Error—ATE - and Relative Pose Error—RPE) and stability. Evaluation of different hardware configurations in simulation results in cost-efficient and more robust planning and evaluation of real world autonomous robots and systems without a need of testing them in real-life environments (at least in the early stages of development). Moreover, compatibility of a simulation with ROS allows parameter tuning and easy deployment of evaluated configurations in actual devices. As an additional result of our study, we provide a description of a general approach together with tools and procedures that can be used to find the best sensor setup in simulation. We have described our procedure in the form of a list so that other researchers can easily use it as a guideline to test their solutions in simulation.

## Related work

The topic of Indoor mapping and localization has attracted considerable interest in recent years. Different types of algorithms can be used for this purpose, which can operate on data from different sensors (usually cameras or LiDARs). LiDARs provide data that is more robust and accurate, as well as less noisy, and sensitive to lighting changes. Therefore, LiDAR point clouds are the most reliable data for the SLAM input^5^. To improve SLAM accuracy, some algorithms apply fusion of laser and visual data^15^. LiDAR-based SLAM can operate on 2-dimensional^16,17^ or 3-dimensional^18,19^ data. In the case of 2D LiDAR-based SLAM, the limited number of dimensions results in inability to provide the estimation of the robot’s pose on uneven ground with six degrees of freedom^20^. Nevertheless, algorithms operating on 2D point clouds are more computationally-efficient than those operating on the 3D ones. Google Cartographer is one of the commonly used LiDAR-based SLAM algorithms. It can operate on 2D and 3D LiDAR data. The alternative is HDL Graph SLAM, which is similar to Cartographer in some aspects (they both use Graph SLAM). The main difference is that HDL Graph SLAM does not use IMU data to estimate odometry, but uses LiDAR data for this purpose. In^21^ it was proven that its accuracy significantly degrades with time. Authors were also unable to obtain a coherent map of the environment. It operates only using 3D Point Clouds. RTAB-Map (Real-Time Appearance-Based Mapping)^19^ strongly depends on camera data (that we decided not to use) to deal with loop closure effect^22^. Other LiDAR-based SLAM algorithms include Normal Distribution Transform (NDT) SLAM^23^ and LiDAR Odometry and Mapping (LOAM) SLAM^11^. In^13^, the comparison of these algorithms with Google Cartographer shows that the Cartographer obtains the best efficiency. Also in work^14^, it has been shown that Google Cartographer significantly outperforms other tested algorithms. Taking all of these research works into consideration, and also the fact that Google Cartographer can operate based on a wide spectrum of sensors, we decided to use this particular algorithm in our study.

Due to the importance of SLAM algorithms, research papers can be found, which compare different SLAM methods and systems in terms of mobile robotics indoor navigation and mapping technologies. In^24^ they compare four ROS-based monocular SLAM methods: REMODE, ORB-SLAM, LSD-SLAM and DPPTAM. It was proven there that these visual SLAM algorithms can successfully detect large objects, obstacles and corners, however they had difficulties with detection of homogeneously colored walls (common in indoor environments), which strongly limits applicability of monocular SLAM-related techniques for indoor mapping and navigation. In^25^ different ROS-based visual SLAM algorithms were tested. They compared the obtained trajectories using data from different sensors: a traditional camera, a LiDAR, a stereo camera and a depth sensor. Trajectories were determined by monocular ORB-SLAM and DPPTAM, RTAB-Map and stereo ZedFu. Again, it was proven that visual SLAMs have problems with flat monochrome objects detection. Other papers dealing with visual SLAM comparison are^26–28^.

Other works focus only on LiDAR-based SLAM algorithms comparison, e.g.^14,29–33^. In^29^ they compared the following 2D ROS-based SLAM techniques: CRSM SLAM, Gmapping and Hector SLAM on a Roomba 645 robotic platform. They used an RGB-D Kinect sensor to emulate a laser scanner. In^30^ they conducted experiments with : Gmapping, HectorSLAM, KartoSLAM, LagoSLAMa and CoreSLAM. They compared these algorithms in 2D simple simulations (in ROS Stage) and real world experiments. The simulation assumed no noise added to sensor data and a simple environment with a few walls only. They discussed strengths and weaknesses of each tested solution. In^31^ they compared the following 2D SLAM libraries available in ROS: Gmapping, Google Cartographer and Hector SLAM. In this work, maps constructed using these algorithms were evaluated against the precise ground truth obtained by laser tracker in a static indoor space based on average distance to the nearest neighbor. The obtained results have shown that almost in all cases Google Cartographer obtained the smallest error while generating maps relative to the ground truth presented by a laser tracker. In^33^ they compared the performance of three mapping systems (Matterport, SLAMMER and NAVIS) in two different indoor environments and provided the discussion on advantages and disadvantages of these systems. In^14^ they compared both the LiDAR-based and monocular camera-based SLAM algorithms. Regarding 2D lidar-based algorithms, they tested GMapping, Hector SLAM and Google Cartographer. The results have shown that out of LiDAR-based systems, Google Cartographer demonstrated the best performance and the biggest robustness to environmental changes.

Papers present in the literature regarding the comparison of SLAM algorithms include comparative studies of different SLAM systems (here the necessity to purchase several entire hardware platforms occurs). The advantage of such works is rather impartial evaluation of off-the-shelf solutions for mapping indoor environments. On the other hand, the disadvantage is that there is often a need to add an autonomy system to an already existing robot, rather than providing a ready-made system. Other works compare different SLAM algorithms, making it often necessary to build different hardware platforms and purchase a large number of sensors, as the solutions are compatible with different physical devices. Their advantage is the evaluation of the effectiveness of different algorithms under the same or similar conditions. However, a big disadvantage is that this type of approach will not work when, for example, a set of possible sensors is given in advance and the aim is to test an optimal subset of them. Our solution focuses on creating a method to test and evaluate the performance of the SLAM algorithms using different sets of sensors (2D and 3D LiDARs, IMUs, wheel odometry sensors) to be mounted on a real room decontamination robot. SLAM algorithm taken into consideration is Google Cartographer. This SLAM algorithm turned out to be the best one among the tested algorithms in the 2 papers described above, so the analysis of its different hardware configurations is valuable and, as far as we know, has not been previously covered in the literature.

Papers regarding the usage of simulation for LiDAR-based systems evaluation are limited. In^32^ they used Gazebo simulator to test the performance of navigation of an autonomous golf cart. They deployed numerous sensors in their model (reflecting the actual vehicle): LiDARs, GPS, Camera, IMU, wheel encoders and sonars. Although this use case regards the outdoor environment, it is one of the few works using more realistic simulation for performance evaluation (than e.g. ROS Stage), in this sense it is similar to our approach. Gazebo provides simplified sensor data without taking noise into account and allows very limited control over the operation of these components (which makes tuning parameters using simulation impossible). Although, Gazebo can undoubtedly be used for preliminary tests of robot localization and navigation, it cannot be used for comprehensive evaluation of SLAM algorithm performance. The discussion regarding the usage of different simulation platforms for the purpose of SLAM algorithms performance evaluation can be found in^12^. These platforms (CARLA^34^, AirSim^35^ and LiDARsim^36^) focus mainly on realistic scene generation and data labeling for the purpose of object recognition and not on the creation of realistic raw sensor data. They also consider mainly outdoor, urban environments for the purpose of autonomous cars testing and not the indoor environments (hospitals, airports and supermarkets), in which Location-Based Services are also crucial.

## System overview

### Real system to be modeled

The robot considered in this paper is a remotely controlled robot designed for decontamination of indoor spaces (e.g. hospital rooms). The main task was to develop an autonomy system for this robot, its evaluation and future deployment. The robot uses powerful UV-C lamps, which aim to neutralize viruses (including SARS-CoV 2), bacteria and other microorganisms. By matching the power of the UV lamps and the speed of the robot, surface decontamination can be carried out more efficiently and safely while maintaining high performance.

The implementation of the autonomous driving system for decontamination began with an analysis of the requirements and environmental conditions of the robot. Operating in narrow corridors, among beds in hospital rooms or around chairs in office spaces requires high accuracy and precision of the vehicle localization and mapping system, and also introduces constraints on the size and shape of the robot. In order to ensure high performance and accuracy of the system, it was decided to test different configurations of sensors mounted on the robot. The following devices were considered:

- Three low-cost 2D LiDARs positioned to achieve a 360 degree field of view
- One 3D LiDAR positioned on top of the robot
- High rate IMU
- Encoders placed in the BLDC motors that control the wheels of the robot, allowing the calculation of the odometry.

In Table 1 the parameters of these sensors have been summarized.

**Table 1:** Technical specification of actual sensors used in the study.

**LiDAR 2D RPLIDAR A2M8**	
Range	12 m
Wavelength	785 nm
Field of view	360 deg
Angular resolution	0.9 deg
Range error	< 1% of the distance
Max. measurement rate	8k points/s
Scan rate	10 Hz
Sample duration	0.125 ms
**LiDAR 3D Velodyne VLP-16**	
Range	100 m
Wavelength	903 nm
Field of view (Horizontal)	360 deg
Field of view (Vertical)	30 deg
Channels	16
Angular resolution (Horizontal)	0.25 deg
Angular resolution (Vertical)	2.0 deg
Range accuracy	+/− 3 cm
Max. measurement rate	8k points/s
Scan rate	10 Hz
Sample (1 firing sequence—all 16 lasers) duration	55.296 us
**IMU**	
Gyroscope/Accelerometer data rate	100 Hz
Gyroscope RMS noise	0.06 deg/s
Accelerometer RMS noise	0.06 m/s^2^
Gyroscope dynamic range	+/− 2000 deg/s
Accelerometer dynamic range	+/− 8 g
**BLDC motor**	
Voltage	24 V
Power	250 W
Control	Three-phase, trapezoidal commutation
Encoder	Hall effect sensor

The basis of the experiments was a system with three 2D LiDARs, due to the relatively low cost of these devices compared to 3D LiDARs (which is extremely important for future production of the system under consideration and other practical solutions, both the scientific and the commercial ones). In practical solutions it should be taken into account that adding more sensors involves costs, so available configurations should be analyzed in order to select the optimal one in terms of performance and number/types of sensors. Performing this type of analysis in a highly realistic simulation means that such an analysis can be carried out without having to purchase all the components in the early testing stages. In addition, by creating sensor models that correspond to their real-life counterparts, it is possible to tune the parameters of autonomy algorithms and the operation of these sensors in simulation, which can significantly accelerate and facilitate the development and deployment process.

### Google Cartographer SLAM algorithm

Google Cartographer is a system responsible for simultaneous localization and mapping in both 2D and 3D and also across multiple platforms and different sensor configurations^9^. It is a platform that is suitable also for systems with limited computational resources. Its main source of data are LiDARs (both 2D and 3D LiDARs can be used). Other data can be added to potentially improve the SLAM accuracy: odometry pose, IMU data (which delivers information regarding angular velocity and linear acceleration) and fixed frame pose. Two types of SLAM are used by the system: Local and Global SLAM^9^. Local SLAM is responsible for scan matching (optimization with Ceres Solver^37^), motion filtering, sub-maps and current trajectory creation. The information generated by Local SLAM goes to Global SLAM, in which the following tasks are performed: matching the sub-maps generated by Local SLAM and creation of a global map^10^, loop closure detection and correction of a global map when loop closure is detected^11^.

To build the map, Cartographer uses the projection of successive laser scans onto a 2D plane. To effectively estimate the position of successive scans, especially in the case of unstable platforms^38^, IMU is used to estimate the gravity vector. Furthermore, based on the angular velocity measured by the gyroscope embedded in IMU, it is possible to estimate the rotation value between successive lidar scans. In the absence of IMU, it can also be obtained using a more rigorous and accurate scan matcher at the expense of the computational complexity of the SLAM algorithm^38^. However, the computational power requirements for 3D lidar usage make it mandatory to use IMU to properly determine the position between successive scans.

### Modeling of the components and simulation

**Figure 1:**
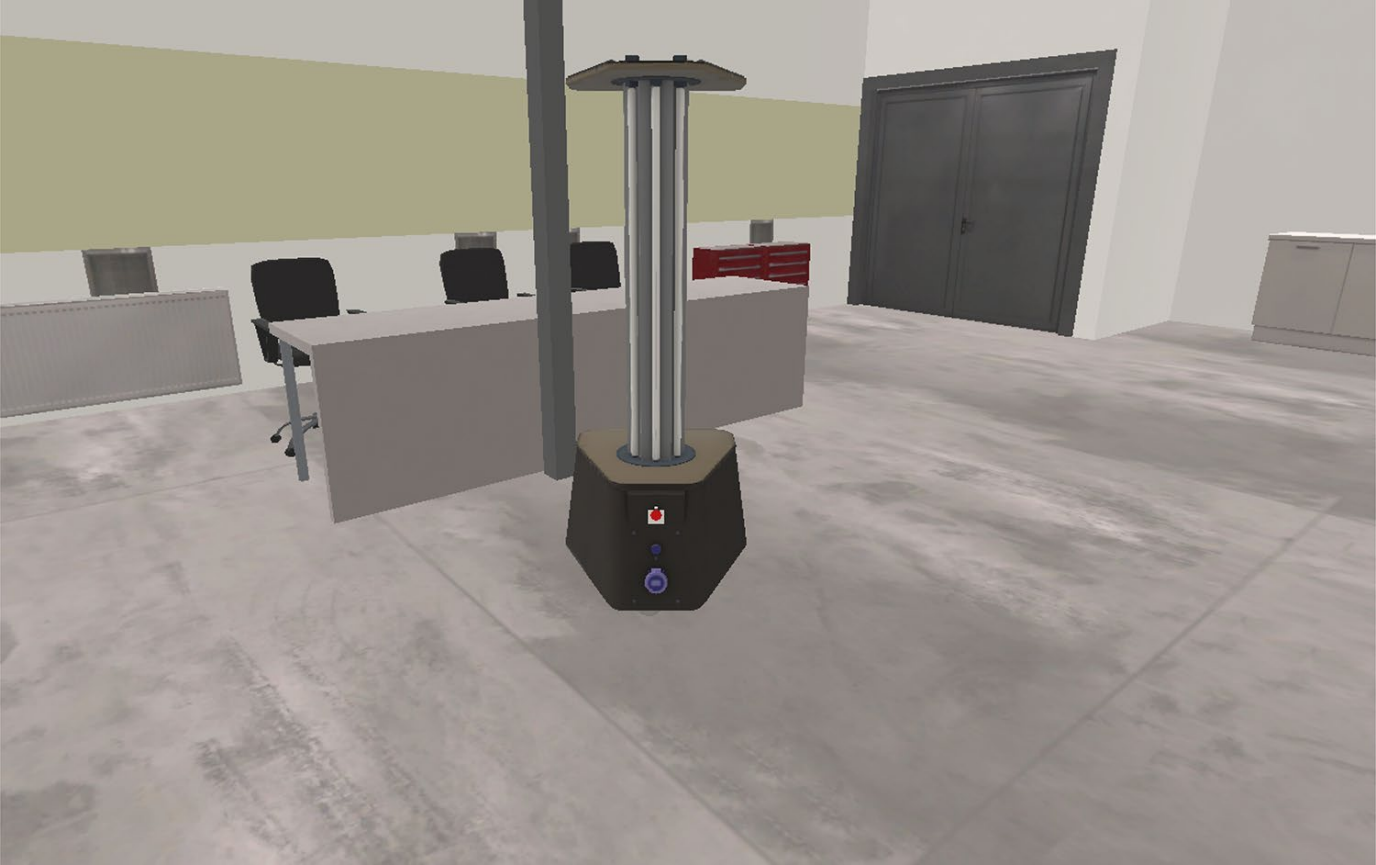
A screenshot from the simulation environment presenting the modeled robot.

To achieve a high-quality simulation, it was necessary to create a highly accurate model of the robot (see Fig. 1) and all the sensors under test. Other available simulations do not place such a priority on accurate simulation of the raw sensor data, which is crucial for meaningful evaluation of the SLAM system in the simulation. Not taking sensor-specific phenomena and errors into account may (very likely) result in the inability to perform an objective evaluation in the simulation. Therefore, in our simulation we put great emphasis on the correct simulation of data generated by 2D and 3D LiDARs (our LiDAR simulation is described in detail in the article^12^, in which we describe the effects present in real lidar data, for example rolling shutter effect, and comprehensively evaluate the accuracy of this simulation), IMU and wheel encoders.

Values that describe sensor-specific quantities along with the measurement time are sent via a predetermined protocol to ROS, where they are packed into standard ROS messages of type sensor_msgs/sensor_type. The data is sent in the same way for IMU, odometry and laser scan data. These data samples are then subscribed by the ROS node Cartographer, which executes the SLAM algorithm. In the case of actual 3D LiDARs, e.g. Velodyne LiDARs, they deliver data in small packets (and not the whole point cloud at once). Cartographer takes these small packets as input and utilizes them to build a full 360 degree scan by calculating the translation of the smaller parts of the scan usually based on IMU data. Due to the high frequency of simulated data generation, the LiDAR frames are accumulated (parameter num_accumulated_range_data) in a similar way as it happens in real life and combined into a larger one by the Cartographer^12^.

Simulation of IMU for the purposes of integration into the SLAM algorithm requires the delivery of acceleration and angular velocity of the object in 3 axes. With the definition of acceleration as the second derivative of the path after time, the change in position of the simulation object between two points in the time period corresponding to the frequency of the real sensor is determined, and then, based on the determined velocity and the previous value, the acceleration is calculated. The final step is to take into consideration the value of the ground acceleration. Similarly, the angular velocity of the object is determined, i.e. the change in the distance travelled in a given time segment is utilized. This is supplemented by the quaternion of the object’s rotation, which is read directly from the simulation. Then, all determined values must be burdened with an error, the value of which is a pseudo-random number from the range of values provided by the manufacturer of a given sensor model or any that can be determined by empirical testing of a real device.

The odometry data used for the SLAM algorithm in Cartographer consists of two components: a pose and a velocity vector, and the corresponding covariance matrices. These values can be calculated, for example, from input data from wheel encoders that count motor revolutions. In our case, each of the two BLDC motors embedded in the wheel is equipped with Hall sensors that inform about the rotor position and a digital output that signals (with the rising edge of a 5V pulse) that the wheel has rotated by 1 degree. These pulses are then counted by the motor controller using 64-bit counters, whose values provide the input for the odometry algorithm^39^.

The simulation of odometry data can be performed at different levels of abstraction, depending on the desired results. Thus, the position and velocity vector of the vehicle can be read out from the simulator. The alternatives are the aforementioned pulse counters, or even the digital pulses themselves or data from Hall sensors monitoring the position of the rotor. In order to obtain more realistic results, we decided to simulate the encoder counters by calculating the difference in angle between the wheel rotation in successive steps of the physics engine simulation. Then, when the difference reaches a value compatible with the angular resolution of the real encoders, the counter corresponding to the wheel in question is incremented or decremented depending on the direction of movement, and the counter value itself is sent to the autonomy system at a frequency compatible with the controller used. This way, the odometry data could be calculated by the same algorithm that is used on the actual robot.

## Materials and methods

### Experimental environments

In our study, we tested Google Cartographer in two experimental environments: a laboratory room and a hallway. The second scenario represents a larger building with hallways—in such environments loop closing needs to be performed. Both of these environments are the indoor ones, due to the fact that the tested robot’s purpose (room decontamination) is purely indoor.

We first modeled the actual laboratory room (medium-sized room with dimension ~13 $\times$ ~14 m) in our simulation framework (the photo of this actual laboratory can be found in^12^) to conduct the experiment in a realistic simulated environment. Comparable experimental environments have been used in other works aiming to compare different SLAM solutions. E.g. in^25^ the authors used a marked trajectory, of length 11.5 m, inside a typical office-style indoor environment. The room itself was slightly longer than the trajectory. In^14^ the experiments have also been conducted in a medium-size office space. For robots that have to work in different environments (e.g., in industry—in large production areas), we recommend building a larger experimental area (adequate to the application). In our case, due to the application of the robot and the fact that similar experimental environments have been used in the literature, we limit our setup to a smaller environment. Such a scene, although relatively small and simple, can be perceived as representative for indoor environments.

**Figure 2:**
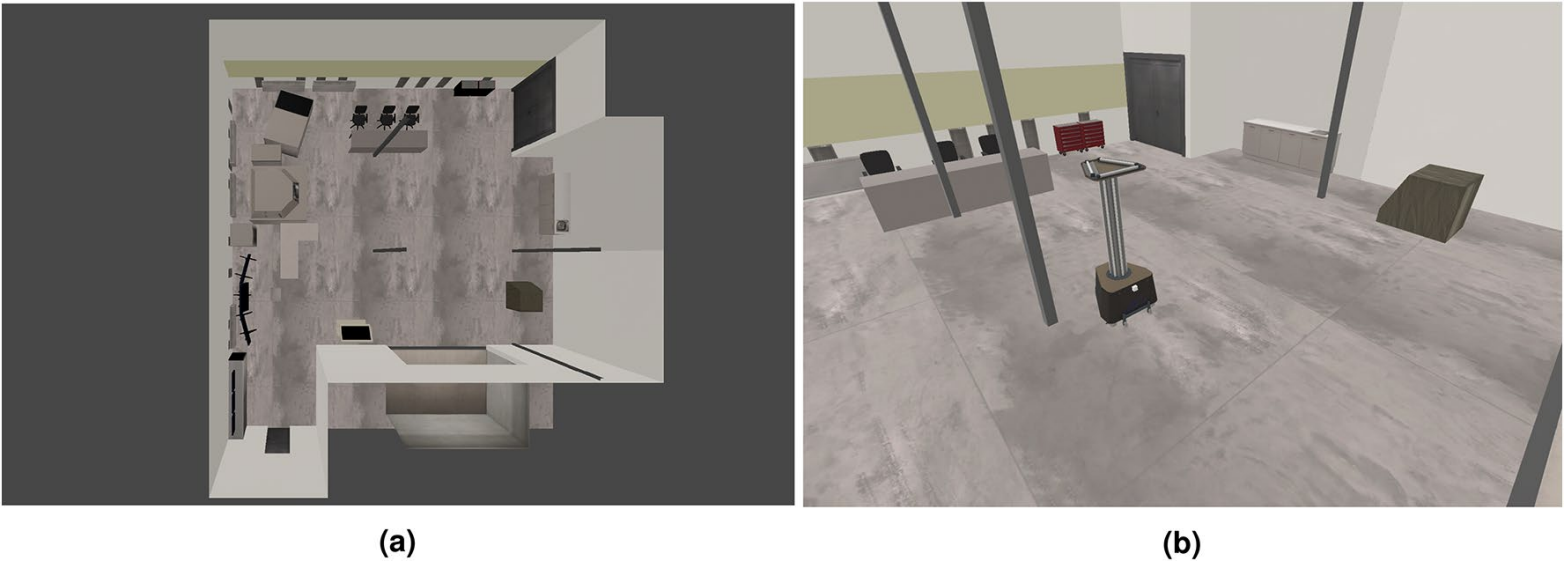
Simulated laboratory room.

In our experimental environment, all of the items and furniture from the actual room were reproduced in the simulation. This way, the experimental environment takes into consideration real furniture, equipment and objects present in this type of space. Furthermore, the elements used make the test environment diverse and relatively complex. This approach minimizes the risk of creating an overly simple experimental environment that would artificially enhance the results obtained and make the analysis results unreliable. The room is roughly rectangular, but not perfectly—it has some recesses. In the room there are, among others, a monitor stand, various types of laboratory machines, cabinets, tables and chairs. There are also several pillars in the room. Figure 2 presents the screenshot from the simulation and the top view.

**Figure 3:**
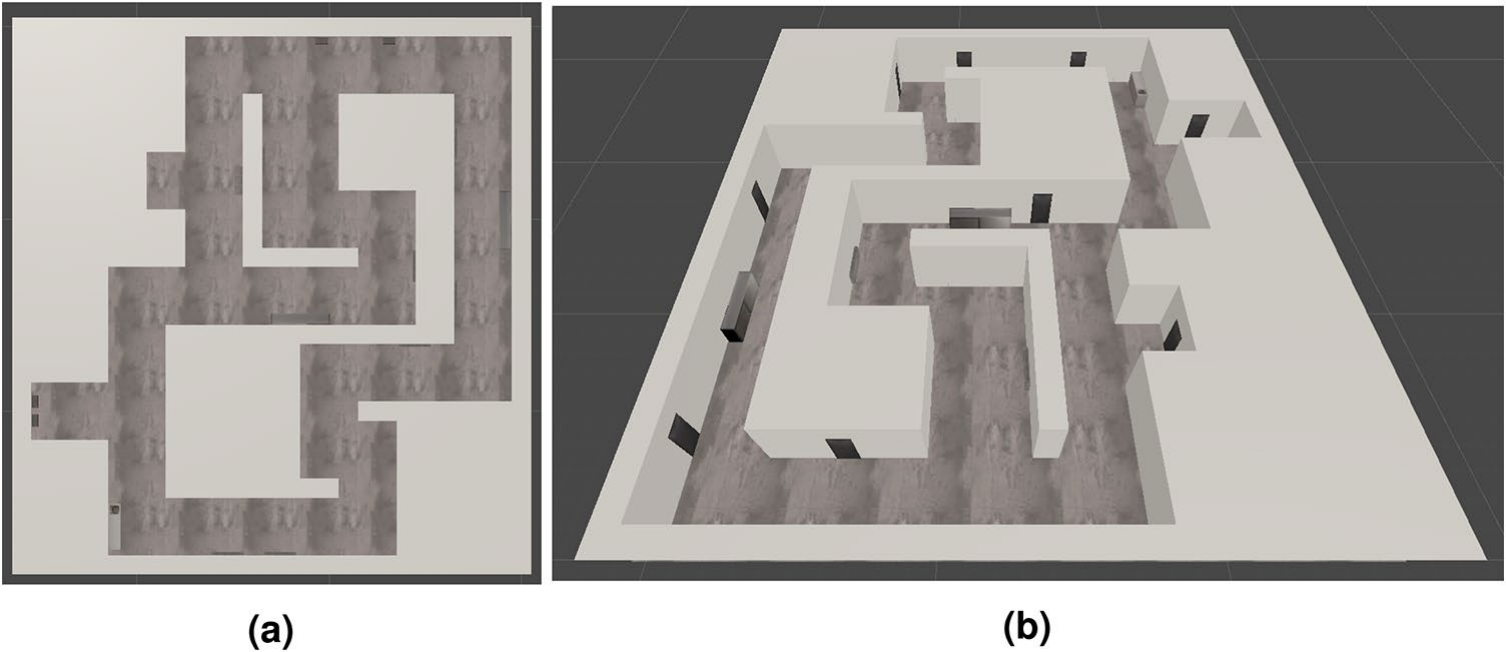
Simulated building with long hallways.

Our second experimental environment—a building with long hallways represents a more challenging scenario—the experimental track is much larger (approximately 70 m long, the size of the whole examined floor of the building is 27 × 24 meters) and it includes long straight segments (without any turns). Figure 3 presents the screenshot from the simulation and the top view.

**Figure 4:**
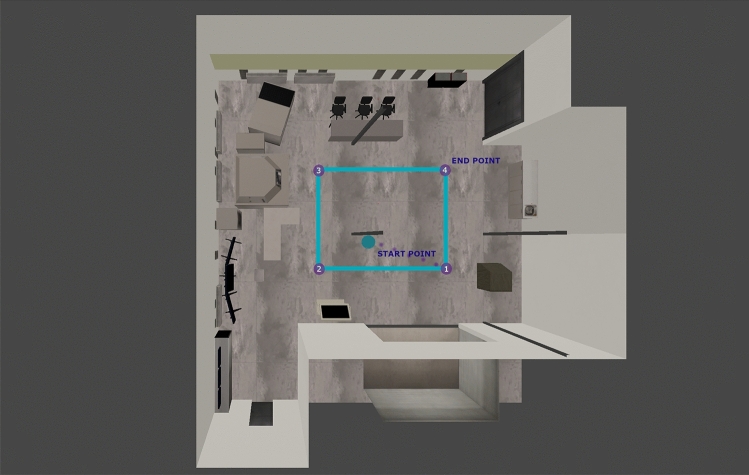
View from the top of the simulation environment with the track ground truth.

To conduct the experiments, we used a model of an actual mobile robot with different hardware configurations. In terms of the first experimental environment, the robot was tasked to drive from the start point to the end point 5 times passing through each control point (the control points were located at the vertices of the square). Track is an example of a closed-loop trajectory consisting of a square. Figure 4 shows the start and end points of the track and the ground truth (the perimeter of the square) used to evaluate the localization performance. Regarding the second experimental environment, our robot was tasked to drive the hallway around and back to the starting point (placed at the bottom left part of the track in Fig. 3a). The simulated trajectory of the robot is the ground truth and is read directly from the simulation which greatly facilitates the measurement of the actual ground truth, which can be troublesome in the real world (it does not require the use of any additional measuring devices that themselves introduce some measurement errors).

During the experiments, the task was to build a map of the surroundings and to estimate the trajectory. On the basis of the results obtained, the quality of the built maps (for the laboratory room case, as it includes much more objects) and the accuracy of trajectory estimation, which includes Relative Pose Error (RPE) and the Absolute Trajectory Error (ATE) have been evaluated (based on the estimated position and the ground truth discussed in the section).

### Evaluation of localization accuracy

Trajectory estimation was assessed using two standard metrics of SLAM accuracy evaluation: Relative Pose Error (RPE) and Absolute Trajectory Error (ATE)^40^. ATE evaluates the absolute pose differences of the estimated and true trajectory, while RPE is determined based on the difference between the estimated and the true motion.

To express the motion of a rigid body, one can use sequences of poses from the actual trajectory (ground truth) $Q_1, Q_2, ..., Q_n \in SE(3)$ and the estimated trajectory $P_1, P_2, ..., P_n \in SE(3)$, where *n* is the number of measured poses. To express *SE*(3) a pair (*R*, *t*) can be used ($R \in SO(3)$, $t \in \mathbb {R}^3$). A Homogeneous matrix *M* can be used to represent a given transformation. Such a transformation takes the following form:

1 $$\begin{aligned} M = \begin{bmatrix} R &{} t \\ 0 &{} 1 \end{bmatrix} \end{aligned}$$

Absolute Trajectory Error measures the global consistency of the estimated and true trajectory. It compares absolute distances between these trajectories. The examined trajectories can be defined in arbitrary coordinate systems. In the case of different coordinate systems, scaling of trajectories is necessary (using a transformation *S*, which can be found using the Horn method^41^). ATE at time step *i* can be deterimed using the following formula^40^:

2 $$\begin{aligned} F_i = Q_i^{-1}SP_i \end{aligned}$$

Then the translational component $trans(F_i)$ can be used to determine the meaningful statistics of norm $|| trans(F_i)||$, e.g. mean or maximum.

Relative Pose Error, on the other hand, can be used to measure the local accuracy of the trajectory over a time interval $\Delta$. It describes the drift of the trajectory and can be used to evaluate the SLAM accuracy at loop closures. RPE at time step *i* can be defined as follows^40^:

3 $$\begin{aligned} E_i = (Q_i^{-1}Q_{i + \Delta })^{-1}(P_i^{-1}P_{i + \Delta }) \end{aligned}$$

In the case of RPE both the translational and rotational component can be used. In our article, we decided to use only the translational part—$trans(E_i)$ (usually it is perceived to be sufficient^40^ for SLAM accuracy evaluation). It can be used to determine the meaningful statistics of norm $|| trans(E_i)||$, e.g. mean or maximum.

Testing in simulation enables easy access to dense and absolute true trajectories (ground truth), therefore both ATE and RPE can be successfully used for evaluation. Both the ATE and RPE measures have been determined based on the simulation (ground truth) and the SLAM results (estimated track). The comparison of the simulator position with the ground truth was performed by sending the current position determined by SLAM to the simulator, which matched the received value with the actual position reading of the robot in the simulation. SLAM computes the current position every 5ms and the current position is sent to the simulator every 100 ms. When a new position arrives, the ground truth is measured in the simulation and they are both saved to a csv file with the results. Such measurements were possible due to the synchronization of the starting position of the SLAM algorithm with the spawn point of the object in the simulation. We decided to sample the robot’s position and calculate the error as frequently in order to minimize the impact of the outliers on the results, thus making them as reliable as possible.

**Figure 5:**
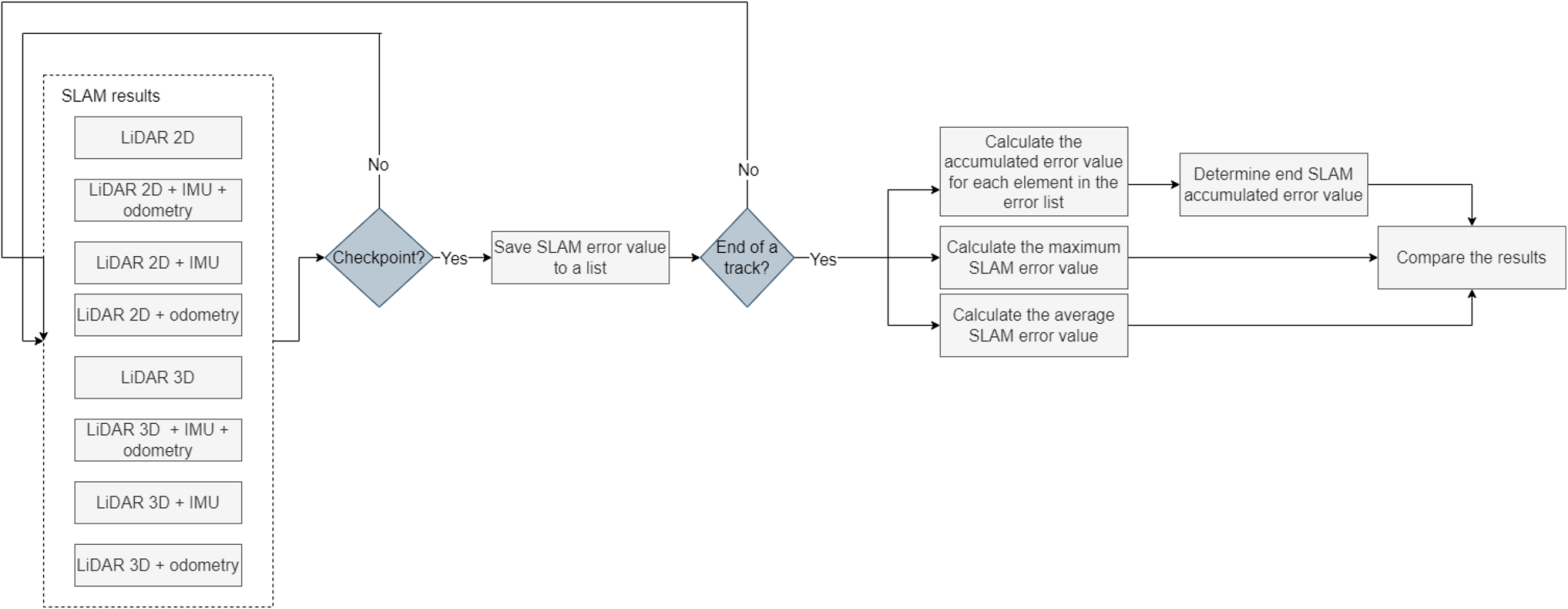
Evaluation procedure of localization performance.

Based on the gathered data, we calculated the following meaningful statistics of ATE: mean, maximum and end value for the laboratory room. In the case of our second environment we focused on mean and maximum values of ATE. In Fig. 5 we have presented the overview of the methodology used to examine the localization accuracy (represented by ATE). For RPE, we calculated the mean and maximum values for both examined tracks.

Using the second experimental environment—the one with long hallways—we examined the performance of Google Cartographer’s loop closure mechanism. For that purpose we performed the same experiments (with eight examined hardware configurations), but we switched off the loop closure mechanism. We compared these results with those obtained for the same scenarios, but with the loop closure mechanism switched on to examine its impact on the overall errors. In our comparison, the RPE results were taken into consideration.

### Evaluation of mapping accuracy

To enable the accuracy evaluation of mapping performance, we used RViz to visualize maps created by Google Cartographer for all hardware configurations. To evaluate the mapping performance, we have split the problem into the following two subproblems:

- General overview and evaluation of key objects
- Accuracy evaluation of key objects representation.

 In the first subproblem we have focused on general features and accuracy of the occupancy grids: we have analyzed the occurrence of map drift and its causes, we have overlaid the occupancy grids obtained via the SLAM algorithm on the simulation screenshots and assessed their overall accuracy in regard to the map from the simulation. We perform the first kind of analysis for both environments.

In the second stage (Accuracy evaluation of key objects representation) we have chosen some characteristic objects from our laboratory room and marked them on the simulation screenshot. We decided to perform the second kind only for the laboratory room environment (it contains more objects with more advanced shapes than the second environment). In our detailed analysis we have taken into consideration the following elements:

- Walls and wall niches
- Objects close to the wall
- Free-standing objects
- Pillars.

**Figure 6:**
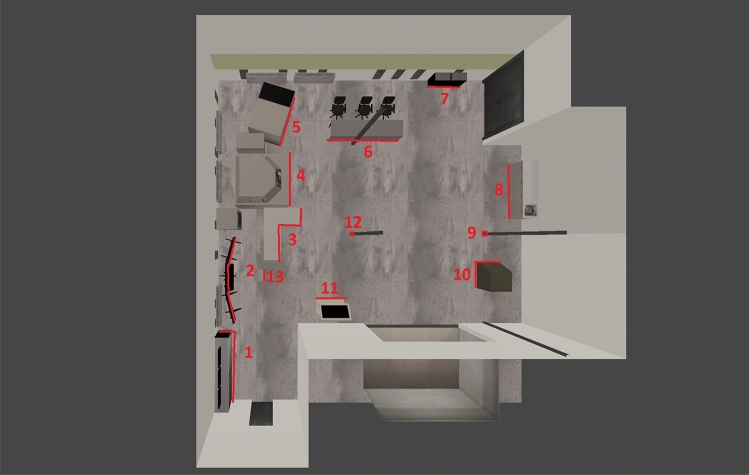
View from the top of the simulation environment with the characteristic objects marked.

 We have assessed the accuracy of representation of these objects in the occupancy grids. The objects present have been marked in Fig. 6.

## Results and discussion

In the article, we have focused on the evaluation of both key aspects of SLAM: Localization and Mapping. The accuracy evaluation of localization can be found in Section “Comparison of trajectory estimation” and the one for mapping—in Section “Comparison of mapping performance”. Section “Comparison of loop closure performance” describes the loop closure performance.

### Comparison of trajectory estimation

**Figure 7:**
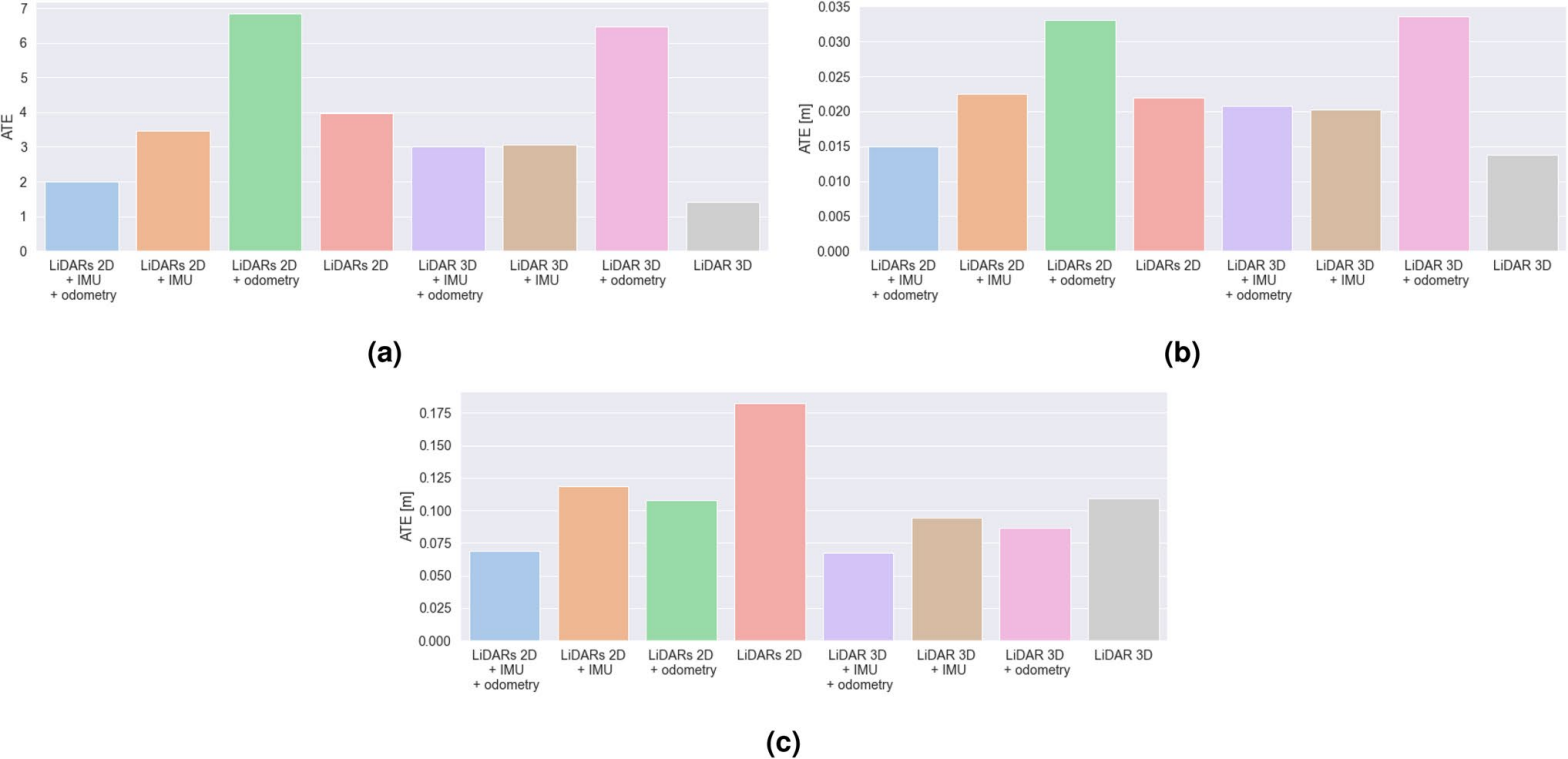
Bar plots representing: (**a**) end, (**b**) average, (**c**) maximum error values (ATE) for different SLAM hardware configurations for the laboratory room environment.

**Figure 8:**
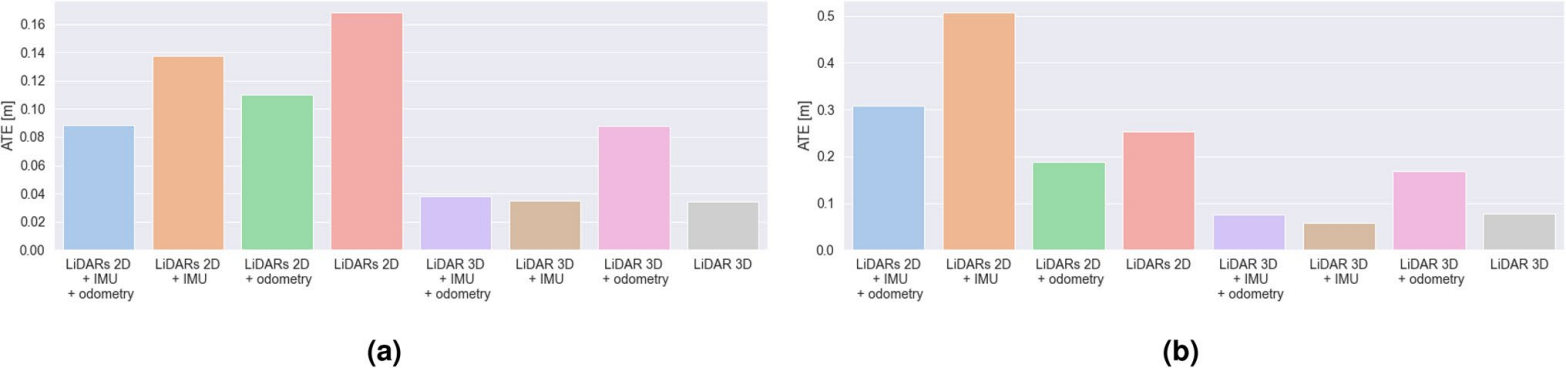
Bar plots representing: (**a**) average and (**b**) maximum error values (ATE) for different SLAM hardware configurations for the environment with long hallways.

**Figure 9:**
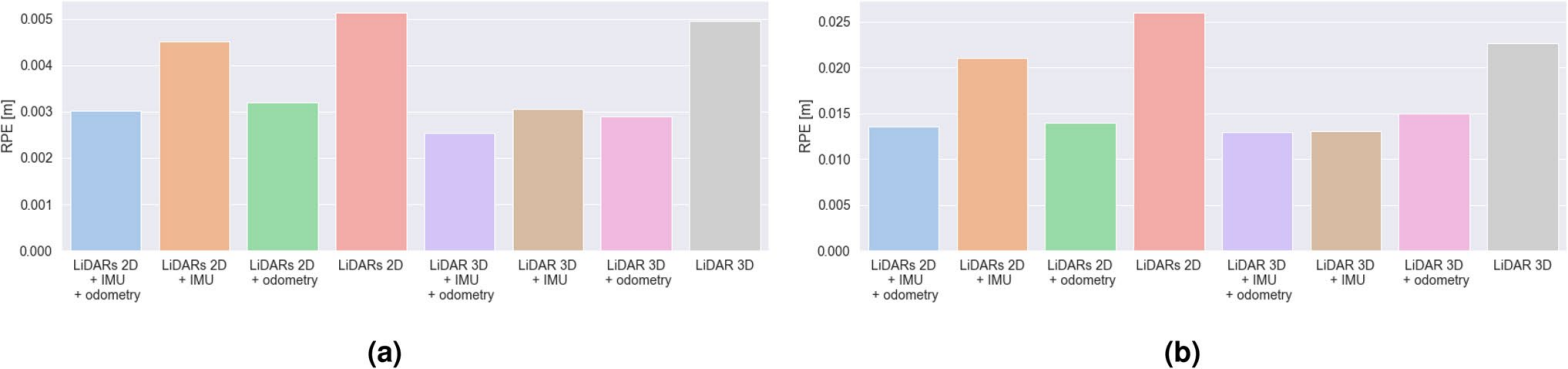
Bar plots representing: (**a**) average and (**b**) maximum error values (RPE) for different SLAM hardware configurations for the laboratory room environment.

**Figure 10:**
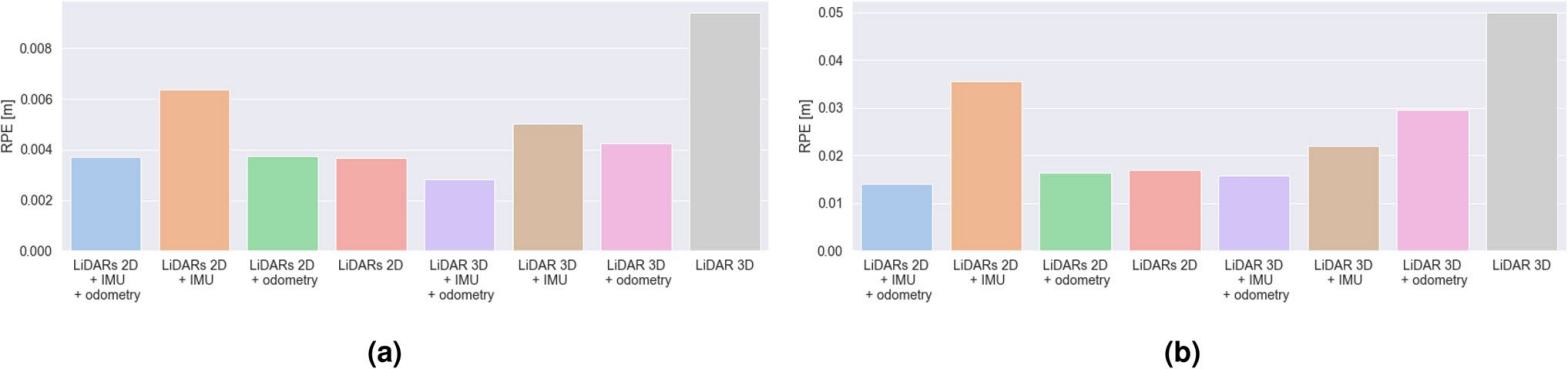
Bar plots representing: (**a**) average and (**b**) maximum error values (RPE) for different SLAM hardware configurations for the environment with long hallways.

We assess the accuracy of trajectory estimation and localization performance of different hardware configurations of Google Cartographer SLAM algorithm based on the results of ATE and RPE measures. We present the results in Figs. 7 and 8. The results obtained in the laboratory room simulation include end, mean and maximum SLAM error (ATE) bar plots presented in Fig. 7 and mean and maximum RPE results in Fig. 9. In the case of the second experimental environment—a building with hallways we focus on mean and maximum ATE and RPE results (Figs. 8 and 10 respectively).

#### LiDARs 2D

In the case of the laboratory room, a relatively low value of the mean ATE was observed, however the maximum value is the highest one out of all examined ones. It can be due to the fact that the estimation is determined only on the basis of the external environment represented by LiDAR data.

For the second experimental environment, the obtained mean and maximum ATE values are higher. In this case LiDARs 2D with no additional sensors provided us with the poorest accuracy. It can be caused by the fact the examined longest hallways have more than 25 meters in length—which is much longer than the range of the LiDARs 2D used (12 m).

In this case, the RPE values are contrasting—for the laboratory room are the highest ones out of all the examined cases, and for the hallway—the determined values (both in terms of mean and maximum value) are close to the smallest ones.

#### LiDARs 2D + IMU

At the beginning of our experiment in a laboratory room, the maximum ATE error is higher than in the case of LiDARs + odometry, however, due to much lower average error values, the final value of error is much lower for LiDARs and IMU.

For the hallway environment, the obtained mean and maximum ATE values are higher. In this case, the mean error is larger than for the LiDARs + odometry case, which can be caused by the fact that the odometry can improve the SLAM performance on long straight tracks (in the first case, the turns, for which the odometry encoders in wheels exhibit poor performance were dominating).

The mean and maximum RPE values are in both cases quite large (although smaller than LiDAR 3D in both cases). Only after the fusion with the odometry data from the wheels a slight improvement over the LiDARs 2D + odometry case can be observed.

#### LiDARs 2D + odometry

In this case, the results show the worst SLAM performance according to mean and end ATE values. It is clearly visible that, although the maximum error is not the highest, the highest value of the mean SLAM error results in fast degradation of the pose accuracy estimation. The main cause of this can be the low accuracy of odometry estimation based on data from encoders in wheels in terms of rotations. The accuracy of these encoders is highly impacted by skidding, driving on uneven surfaces and even slight deformations of wheels. All of these factors can significantly reduce the pose estimation accuracy.

In our second environment, using wheel odometry allowed us to improve the results (ATE) of a pure LiDAR configuration and LiDARs + IMU. It can be caused by the fact that the problems with wheel odometry regard mostly to the rotational movement estimation. Due to the fact that this environment includes more straight tracks than corners and turns, odometry can have a positive impact on the localization accuracy.

In terms of RPE, the results are much more similar (slightly higher mean error for the hallway case). RPE is slightly higher than for the LiDARs 2D + odometry + IMU, LiDAR3D + IMU + odometry and LiDAR3D + odometry configurations. Nevertheless it obtained much smaller mean RPE values than LiDARs 2D, LiDARs 2D + IMU and LiDAR 3D (the laboratory room) and LiDARs 2D + IMU and LiDAR 3D for the hallway.

#### LiDARs 2D + odometry + IMU

The average ATE value in the laboratory room for this case is the lowest one of the all examined cases, and the maximum value is the second lowest value (the difference between this value and the lowest one—obtained for LiDAR 3D and odometry—is only 2 mm). The results show that using data representing the inner state of the robot and the external environment allows us to achieve the highest accuracy over time of the pose estimation.

For the hallway environment, the obtained mean and maximum ATE values are higher than for the same case in the laboratory room, nevertheless the error is still the smallest one out of all configurations with LiDARs 2D. It is clearly visible that the fusion of data from multiple sensors can result in better accuracy of the solution.

Mean RPE values are quite similar for both environments (although approximately 1mm higher for the hallway environment). The main difference is that for the laboratory room, this configuration obtained slightly higher values than LiDAR3D + IMU + odometry and LiDAR3D + odometry. Nevertheless it also obtained a much smaller mean RPE than LiDARs 2D, LiDARs 2D + IMU and LiDAR 3D.

Although the ATE results obtained with LiDAR 3D were slightly better in terms of mean error values (maximum ATE is smaller for LiDARs 2D + odometry + IMU), the RPE values are much smaller for the current case (in fact, only in the case of LiDAR 3D + IMU + odometry we were able to obtain slightly lower mean RPE values in both the environments). Also, this configuration is more cost-efficient than the one with LiDAR 3D. Also, this configuration seems to be the best one in the case of the examined decontamination robot in medium-size rooms (e.g. hospital rooms, laboratories, office rooms), and still sufficient for long hallways (6 cm worse results than the best case, which allows to obtain a very good accuracy).

#### LiDAR 3D

Mean ATE results for the laboratory environment were the smallest ones in this case (although the mean value is only slightly smaller than in the LiDARs 2D + odometry + IMU case). In contrast, the maximum ATE is higher for LiDAR 3D than for the LiDARs 2D + odometry + IMU case. In the case of a second environment, the mean ATE results are very similar, although slightly higher. In both cases, the mean ATE values are the highest ones out of all examined cases.

In terms of RPE, the mean and maximum values obtained are virtually the highest ones out of all the examined cases (in the case of laboratory room, the values are slightly smaller than the worst case—LiDARs 2D). It is clearly visible that in this case, the accuracy of the local trajectory suffers when no additional (inertial) sensors are used.

#### LiDAR 3D + IMU

In the case of the laboratory room, the mean ATE value is higher than in pure LiDARs 3D case. In this case, adding data from this additional source can decrease accuracy in terms of ATE. In the case of our second environment, the obtained mean and maximum ATE are similar to the ones obtained for a pure LiDAR 3D case (slightly worse mean results and slightly better maximum results). In this case the examined configuration obtained better results than the ones based on LiDARs 2D (most probably due to the higher range of the LiDAR 3D used in our study).

In terms of RPE, it is clearly visible that adding data from an additional sensor—IMU—allows us to improve the local trajectory estimation results in both the examined cases. In the case of a laboratory room, the mean accuracy is similar to the LiDARs2D + IMU + odometry, however in the second environment it is the LiDARs2D + IMU + odometry that performs better.

#### LiDAR 3D + odometry

Mean ATE value in our laboratory room environment was the second highest one out of all the examined configurations, and in the second case—the highest one out of all the examined 3D LiDAR-based configurations. It can be caused by the poor accuracy of the wheel odometry sensors (especially for rotation).

In terms of RPE, it is visible that, also in this case, fusion with data from an additional sensor—odometry wheel encoders—can improve the local trajectory estimation results in both the examined cases. The improvement is not as significant as in the LiDAR 3D + IMU case, but still allows to decrease the error by up to 7 cm.

#### LiDAR 3D + IMU + odometry

In both tested environments, the mean ATE results are very similar to the ones obtained for the LiDAR 3D + IMU case—adding data from two additional sources can decrease the accuracy in terms of ATE in comparison to the pure LiDAR 3D case.

Mean RPE values obtained in both the examined environments, on the other hand, show that data fusion with two additional sensors (IMU and odometry) can decrease the RPE value more than each sensor used individually. The mean results are slightly lower than the ones obtained for the LiDARs 2D + odometry + IMU case, and similar in terms of the maximum values.

### Comparison of loop closure performance

**Figure 11:**
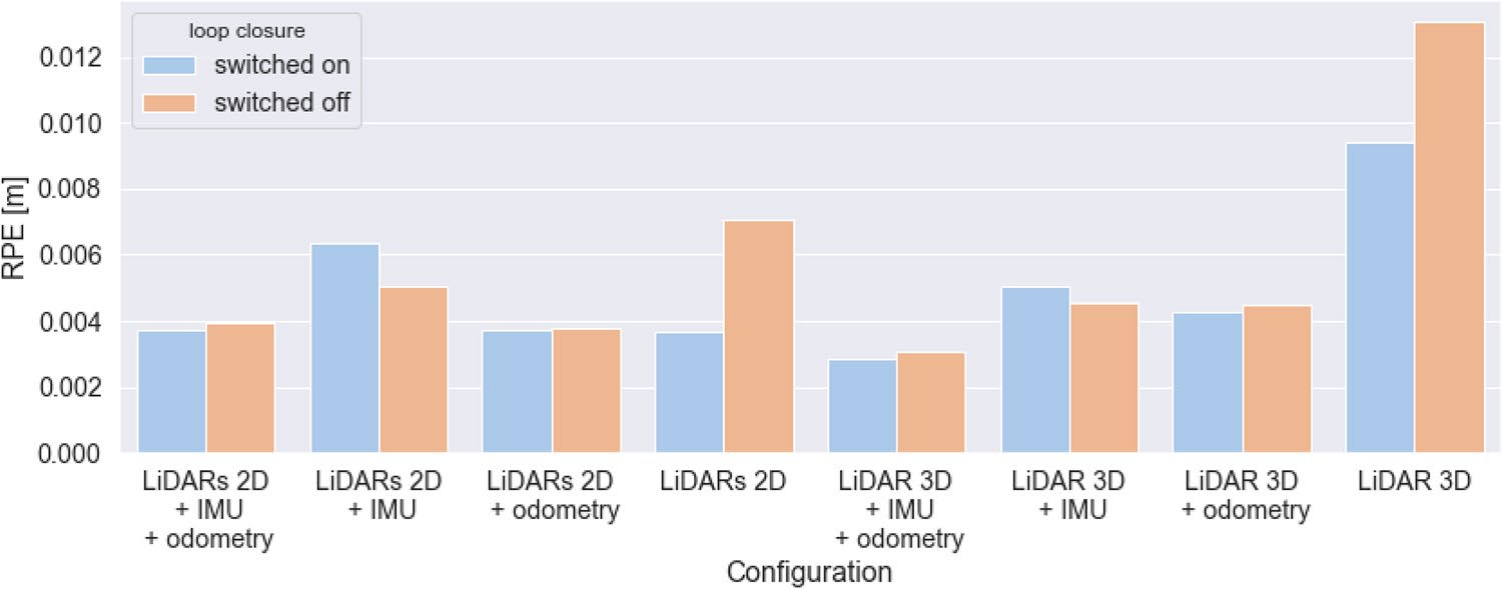
Comparison of RPE values for the examined hardware configuration for the operation in the second environment (hallway) with loop closure switched on and off.

Using our second environment—the building with hallways—we examined the impact of using loop closure mechanism in Google Cartographer. In Fig. 11 we present the comparison of mean RPE values obtained for all the examined configurations with loop closure mechanism switched on and off. It is clearly visible that in all of the examined cases, in which additional sensors were used, the impact of the loop closure mechanism is not as significant as in the case of using only LiDARs (the same for 2D and 3D). It shows that adding data from additional sensors (IMU, odometry) can improve the stability of the SLAM algorithm even without using the loop closure mechanism.

### Comparison of mapping performance

**Figure 12:**
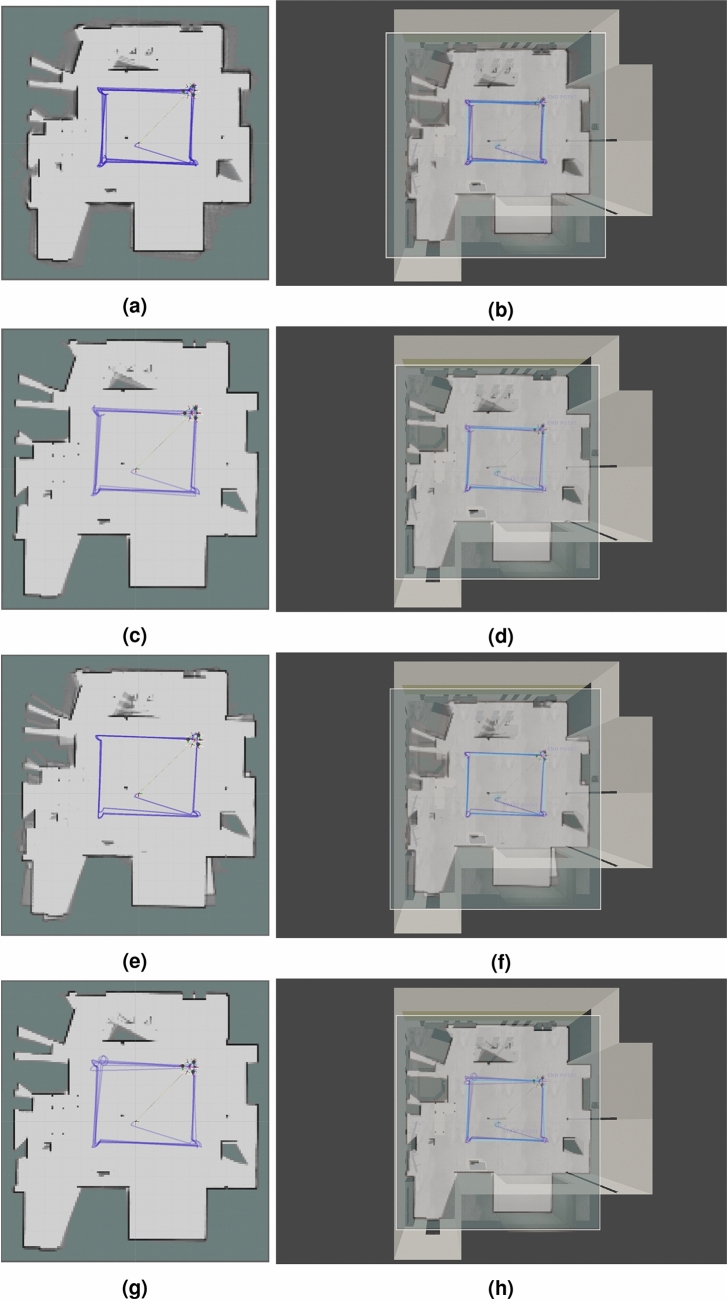
Maps generated with Google Cartographer for the following hardware configurations and maps placed on the screen of the simulated environment: (**a**), (**b**) 3xLiDARs 2D; (**c**), (**d**) 3xLiDARs 2D + IMU; (**e**), (**f**) 3xLiDARs 2D + odometry; (**g**), (**h**) 3xLiDARs 2D + IMU + odometry.

**Figure 13:**
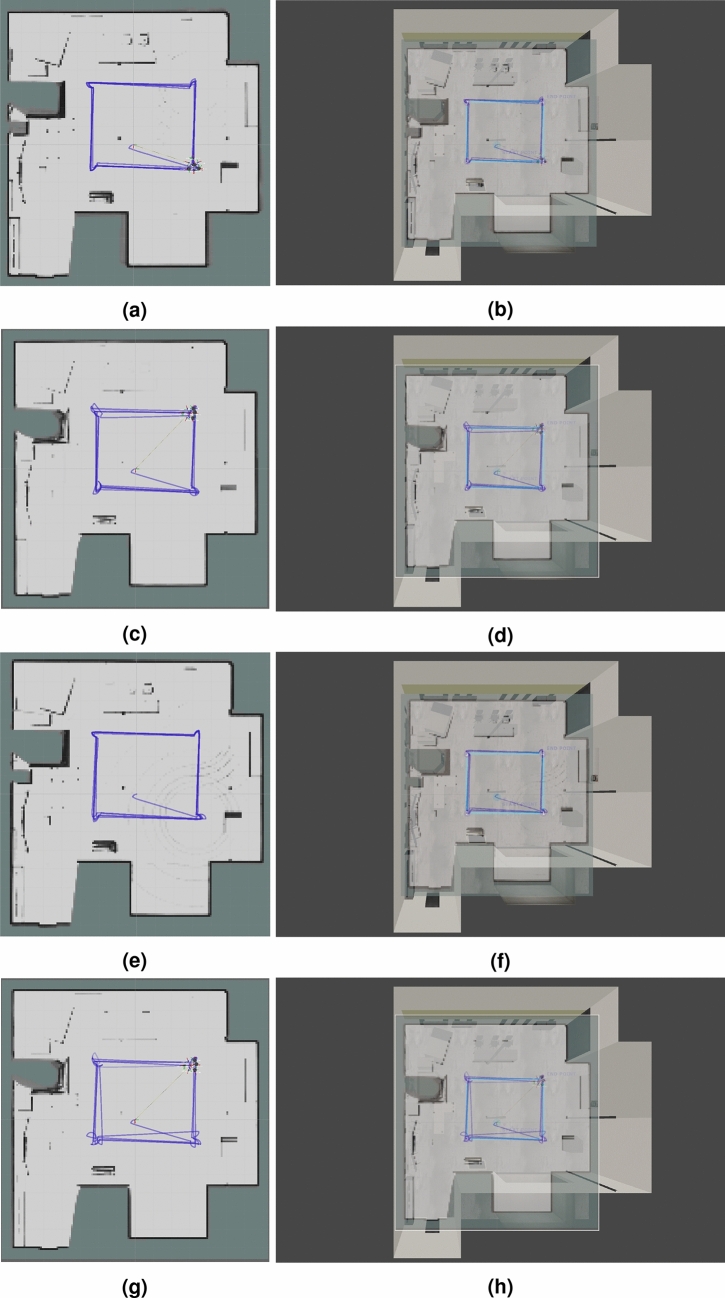
Maps generated with Google Cartographer for the following hardware configurations and maps placed on the screen of the simulated environment: (**a**), (**b**) 1xLiDAR 3D; (**c**), (**d**) 1xLiDAR 3D + IMU; (**e**), (**f**) 1xLiDAR 3D odometry; (**g**), (**h**) 1xLiDAR 3D + IMU + odometry.

**Figure 14:**
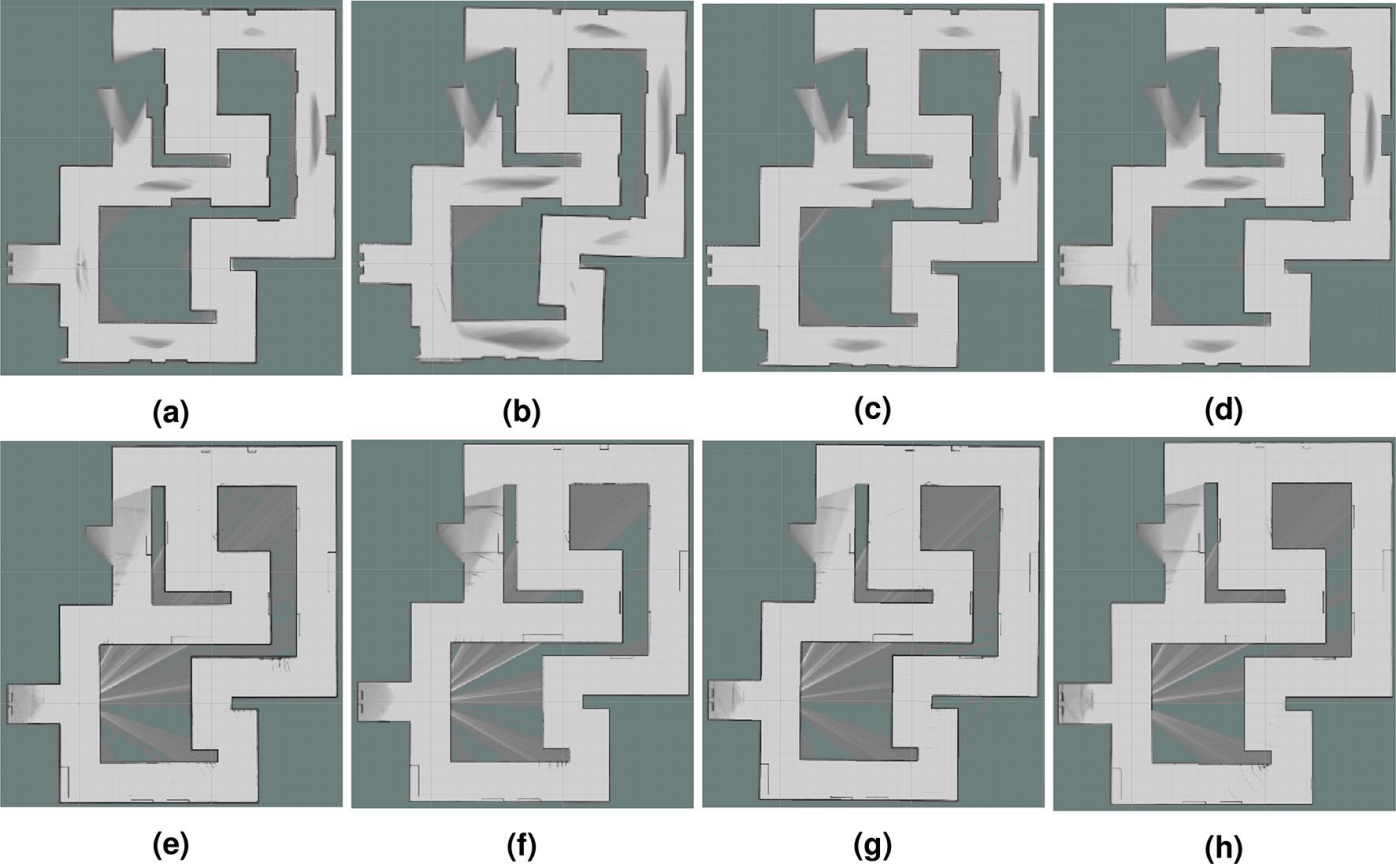
Maps generated for the following hardware configurations—general overview of the hallway environment: (**a**) 3xLiDARs 2D; (**b**) 3xLiDARs 2D + IMU; (**c**) 3xLiDARs 2D + odometry; (**d**) 3xLiDARs 2D+ IMU + odometry; (**e**) 1xLiDAR 3D; (**f**) 1xLiDAR 3D + IMU; (**g**) 1xLiDAR 3D odometry; (**h**) 1xLiDAR 3D + IMU + odometry.

We have used RViz to visualize maps (occupancy grids) and trajectories recovered from Google Cartographer 2D SLAM algorithm. These trajectories have already been evaluated in Section “Comparison of trajectory estimation”. The generated maps are assessed in terms of accuracy of detection of main scene elements (walls and key objects). We present the results in Figs. 12, 13, 14. To facilitate the evaluation and interpretation of the results, for the laboratory room environment, we not only provide the generated occupancy grids, but also the top views of the simulated environment with the occupancy grids layed on top of them (see Figs. 12 and 13). We also chose some characteristic objects in the laboratory room to discuss the accuracy of reflection of these objects in the obtained occupancy grids (see Fig. 6, which we marked and assigned the labels to 13 objects).

#### General overview and evaluation of generated maps

In Figs. 12 and 13, we presented the maps obtained for the laboratory room environment (for setups with 2D and 3D LiDARs respectively). It is clearly visible that for all the examined cases we obtained relatively accurate maps of this environment. All maps reflect the majority of main scene elements: walls, wall niches, a room behind the door frame, pillars and the objects present in the room. It can be observed that occupancy grids obtained in the cases of LiDAR 2D with no additional sensors and LiDAR 2D with odometry suffer from drifts. In the case of SLAM using only 2D LiDARs during operation when turning, accelerating and braking it was visible how the location drifted, but when the vehicle was moving at a constant speed in a straight line the stability returned. This shows that using 2D LiDARs alone is sufficient for simple linear trajectories, but suffers from drifts with rotational motion and changes in linear velocity. Unfortunately in practical cases (mapping, navigation) such constraints are not acceptable. In the case of 2D LiDAR with odometry, map drift is much more visible and occurs when robot rotates. This may be due to inaccurate pose estimation based on yaw velocity calculations from wheel encoder data (odometry data are usually subject to large errors and noise). IMU readings are usually more accurate than those from odometry, which results in the better quality of the resulting map in the 2D case. Using odometry, on the other hand, improves the stability of the trajectory (both for the 2D and 3D cases).

In the case of the hallway environment, our main focus was to examine the performance of the algorithm when the closure of a relatively large loop occurs. That is why our environment is quite simple and contains only simple pieces of furniture. Due to its different purpose, we do not perform a detailed qualitative analysis of the key objects. Instead, we focus on the quality of the general mapping of the constructed map. In Fig. 14, we present the maps obtained in RViz. It is visible that the main difference in quality is the result of using different types of LiDARs. In the case of the single 3D LiDAR, which has a much larger range, the shape of the tunnel is much better reflected. Our root did not include the tunnel branch in the left upper corner—it can be noticed that in this area, the map could not be created using LiDARs 2D (12 meters range). On the other hand, LiDARs 2D can better reflect the simple objects placed along the hallway—radiators, doors, simple cabinets (e.g. see the wall on the top).

#### Accuracy evaluation of key objects representation in occupancy grids of the laboratory room

In the case of our laboratory environment, which includes many heterogeneous objects (furniture, machinery, pillars etc.), we can enhance our overall verification of the generated maps with the evaluation of key objects representation in the obtained occupancy grids. It can be observed that all of the LiDAR 2D cases are not able to detect the walls behind the heavy machinery (see point 5 in Fig. 6) in the left upper corner of maps in Figs. 12 and 13. It is cause by the fact, that the 2D LiDARs due to the shape of the robot are embedded on its case (obviously, LiDARs cannot be installed on the UV lamp), thus the LiDARs are placed relatively low and they the walls under discussion wall are not in their field of view. That is why SLAM algorithm perceives heavy machinery as walls of the room in that region. In the cases with 3D SLAM walls in this region are well reflected, because the 3D LiDAR stands on top of the UV lamp (it is necessary to provide it with a 360 degree view). Nethertheless, heavy machinery in this region is less visible on the occupancy grids (a thin line). Analogous results were obtained for other objects next to the walls (see points 1, 2, 7, 8 and 11 in Fig. 6 and the corresponding regions of maps in Figs. 12 and 13). In these cases, objects are treated as walls. One exception is for the biggest machine in the room—object 4 in Fig. 6—for this case in all of the cases we were unable to obtain a good representation of walls in this region (this object is much higher than the rest, so also in 3D cases the LiDAR was placed to low to accurately reflect this region).

Regarding free-standing objects (see points 3, 6, 10, 13 in Fig. 6), they are much more visible on occupancy grids obtained with LiDAR 2D cases. Even the table (see point 6 in Fig. 6), which is one of the biggest objects in the room is barely visible on occupancy grids for LiDAR 3D cases. In contrast, it is clearly marked in all of the LiDAR 2D cases. The situation is very similar for another big object—point 10 in Fig. 6—which was reconstructed very well for LiDAR 2D cases (slightly worse for LiDAR 2D with odometry) and poorly for LiDAR 3D cases. The most demanding object—point 3 in Fig. 6 (a table with thin legs)—was weakly represented in the occupancy grids of all the examined cases, however for the LiDAR 2D cases it is better visible (legs of the table are visible as dots in the occupancy grid), especially for cases with IMU. The smallest object in the scene—point 13 in Fig. 6 (a small cabinet)—is virtually not visible in occupancy grids of LiDAR 3D cases (a very blurry lines can be observed) and it is clearly visible for LiDAR 2D cases (the best for LiDAR 2D + IMU + odometry).

Thin pillars present in the laboratory room (points 9 and 12 in Fig. 6) can be found on all occupancy grid, but similarly to other objects they are better reflected for LiDAR 2D cases than for LiDAR 3D cases, which shows that not only height (which is important in our case as 2D and 3D LiDARs are place on different parts of the robot) of the object impacts its detection accuracy.

## Discussion of results

In terms of localization accuracy, the LiDARs 2D, IMU and odometry configuration seems to be the best-suited one for the medium-sized rooms (laboratory rooms, hospital rooms, offices). For long hallways configurations based on LiDARs 3D work slightly better in terms of ATE errors, however in both the examined cases they usually obtain worse results for the local trajectory estimation than the LiDARs 2D, IMU and odometry configuration. Taking into consideration the fact that this configuration is more cost-efficient, it seems to be a sufficient configuration for both the examined standard applications for a decontamination robot (or other similar robots used indoor).

Our results show that using additional sensors makes the algorithm more resilient to switching off the loop closure mechanism (both in terms of using LiDARs 2D and 3D as a base for the SLAM algorithm). It shows that adding data from additional sensors, i.e. IMU and odometry, can improve the stability of the SLAM algorithm even without using the loop closure mechanism.

In terms of mapping performance, for the 3D cases, the walls of the room were better represented, and for the 2D cases—the objects. It is clearly visible, that the accuracy of wall reconstruction and object detection is highly dependent on laser scanner/scanners placement on the examined robot (2D scanners were place on the case of the robot and 3D scanner—on the top of the UV lamp, which was the only possible place due to the shape of the robot). Therefore, in practical applications sometimes it may be impossible to find the perfect sensor placement that would reflect all of the objects in the room.

When it comes to additional sensors, the use of IMU allows to improve the driving stability of the vehicle (which results in low SLAM error values) and, consequently, to obtain a map of significantly better quality. Using odometry can lead to decrease in Simultaneous Localization and Mapping accuracy, however when used along with IMU sensors can deliver the highest quality results (both in terms of global trajectory estimation (measured using ATE), and local trajectory estimation described using RPE).

## General approach for finding the best sensor setup

In this section, we shortly describe the steps of the testing procedure to find the best sensor setup for an autonomous vehicle in simulation. We also deliver a short description of tools that can be used in the experiments (simulation frameworks, tools that can be used to build a dedicated simulator and for visualization).

### General simulation testing procedure

In the list below, we gathered all the necessary steps to perform comparison of different hardware configurations for SLAM algorithms in simulation. This list can be used as the basis for the testing procedure.

1. Specification of requirements
  - Specification of environmental requirements
    i. Specifying the size of the environment (small, medium, large, etc.)
    ii. Specifying the nature of the environment (indoor, outdoor, underground, etc.)
    iii. Specifying characteristic objects for the environment (furniture, vegetation, static objects, dynamic objects, etc.)
  - Specification of vehicle requirements
    i. CAD model of the vehicle in real scale
    ii. Specifying the type of drive (differential drive, ackermann drive) and its parameters (wheel size, wheelbase, turning radius, speed and acceleration)
    iii. Marking potential mounting points for sensors on the model
  - Defining a possible set of sensors for testing based on environmental requirements and vehicle capabilities
  - Collection of sensor parameters
    i. Determination of IMU parameters (data sampling frequency, measurement errors of accelerometer and gyroscope)
    ii. Determination of lidar parameters (range, angular vertical/horizontal measurement range, vertical/horizontal data resolution)
    iii. Determination of wheel encoder parameters (number of ticks per wheel rotation, min/max range)
    iv. Determination of parameters of other sensors of interest
2. Defining metrics of interest based on the gathered requirements (the list can be expanded depending on the requirements)
  - Definition of quantitative metrics
    i. Absolute Trajectory Error (ATE)
    ii. Relative Positioning Error (RPE)
    iii. Determination of statistics describing selected metrics
  - Definition of qualitative metrics
    i. Evaluation of the overall quality of the generated map (the general outline of the constructed map is taken into account)
    ii. Qualitative evaluation of the obtained map based on the characteristic points selected on this map (furniture, machinery, doors, etc.)
3. Building the appropriate testing environment
  - Preparing the simulation environment
    i. Preparing a model of the environment according to the collected requirements
    ii. Preparing a vehicle model according to the collected requirements
    iii. Preparing sensor models according to the collected requirements
  - Enabling communication between the simulator and the tested system (in our case, the SLAM algorithm)—in our case, the tested algorithm is run via ROS, and the communication is provided by our UDP Bridge (ROS-simulation communication)
  - Development of data collection mechanisms
    i. Ground truth reading in simulation
    ii. SLAM results reading
    iii. Synchronization of measurements
  - Development of data visualization mechanisms
    i. Visualization of the created maps and trajectories
    ii. Visualization of statistics describing quantitative metrics
4. Defining a test route in a simulation
  - Determining the characteristic points of the test environment for evaluating the quality of the generated maps
  - Planning the route considering the characteristic points of the environment
  - Selecting measurement points along the route (these can be fixed points on the map or data can be collected at specific time intervals)
5. Measurements
  - Completing the test route for each of the selected hardware configurations
  - Reading the determined location and ground truth at checkpoints
  - Recording the data
    i. Saving the created map
    ii. Saving the numerical results
6. Preparing the results underlying the evaluation
  - Determination of positioning errors (quantitative analysis)
  - Visualization of the obtained maps and analysis of the accuracy of the mapping (qualitative analysis)
7. Comparison of the obtained results and selection of the optimal configuration taking into account the obtained results and other factors of interest (such as the cost of a given hardware configuration)
8. For later stages of development it is recommended to carry out verification with real equipment and verify the results obtained in simulation.

### Tools

#### Available simulators

 In terms of simulation tools, one can use available simulators or build their own one. Freely available simulators range from the ones offering simple visual and physical capabilities (e.g. *Stage* (2D) or *Gazebo* (3D)) to the more advanced ones: e.g. *Microsoft AirSim*^35^ or *Carla*^34^. The tool should be chosen according to the needs. For preliminary testing, even the simpler simulators can be enough, and in the later stages of the development, one should shift to the more advanced ones. *Microsoft AirSim*^35^ is dedicated to aerial vehicles testing, but offers one car model. Unfortunately, the sensor simulation comes without sensor-specific noise, therefore the simulation world is far more idealistic than the real one. *Carla*^34^ seems to be the most powerful freely available simulator for autonomous vehicles testing in urban environments. E.g. it offers numerous urban layouts, sensors used in the autonomous cars and dynamic actors. CARLA does not support ground vehicles with differential steering, which is often used by land platforms. CARLA only supports vehicles with the Ackermann-type steering. Another thing is that CARLA uses OpenDRIVE format to describe the environment. This format is characteristic for description of public roads, therefore it is not suitable for indoor and off-road environments. In such environments, it is often a good option to build a dedicated simulator meeting the requirements of the tested use cases.

#### Building a dedicated simulator

 To build a simulation platform, different tools can be used. In our solution, we used Unity rendering engine. It is based on DirectX 11 framework. The nVidia Physx physics engine is used for real-time calculations. As an alternative, different tools can be used, e.g. Unreal Engine or low-level OpenGL API and Vulkan API.

#### Experiments and visualization

 ROS itself is a good platform for gathering the results and for visualization. ROS offers its own 3D visualization widget—RViz^42^. We also used it to present our results. As an alternative, one can use some more advanced visualization tools, e.g. Foxglove^43^ or Webviz^44^.

#### Communication between ROS and simulation

 To enable ROS-simulation communication, a communication bridge has to be used. In our solution, we used a crafted approach based on the UDP protocol, but also an available ROS solution can be used—ROS Bridge^45^. One solution based on Rosbridge is a set of libraries—ROS#^46^. It is based on Transmission Control Protocol—TCP, Websockets and ROSBridge to exchange data between Unity and ROS.

### Advantages and disadvantages of simulation testing

The great advantage of testing the system in the design phase via simulation is that there is no need to purchase sensors to test all the possible configurations and it is possible to automate this process, as well as to obtain identical conditions for each configuration tested. Below, we provide the list of advantages and disadvantages of testing different algorithms and hardware configurations in a simulation.

#### Advantages of SLAM algorithm testing in simulation

- Possibility to perform more robust tests and more tests in general—it is possible to easily automate SLAM accuracy testing against ground truth in a large number of measurement points (which in reality involves laborious and troublesome measurements burdened with measurement error)
- Access to precise ground truth
- Possibility of conducting experiments under perfectly identical conditions in real time (repeatability of tests)
- Either a map corresponding to a real room can be created or your resources can be easily increased—for example by creating a huge production area in the simulation—it is particularly valuable in the earlier stages of manufacturing
- Cost-effectiveness (only a narrowed down set of sensors has to be purchased).

#### Disadvantages of SLAM algorithm testing in simulation

- Building a very accurate virtual environment is time-consuming or impossible. Nevertheless, in some cases it may not be required.
- Modern simulations (especially those modeled in advanced game engines) are very accurate, but we are still dealing with simplified physics, world and sensors’ models. Still in the later stages of production it would be beneficial to invest in real hardware and perform tests (however, using simulation we can significantly reduce the number of necessary tests).

  This list can be used in the decision-making process regarding the product testing in the simulation—whether such a testing is suitable for a particular application or a stage of the development process. Generally, it seems to be especially helpful in the early stages of the product development process and to reduce the number of necessary real-life tests. Here, we would like to highlight that a simulation is never as detailed as the real world, therefore the real-world verification is recommended in the final stages of the development process—before the real-world deployment of any solution. Nevertheless, testing in simulation can greatly reduce the time and cost needed to select the right sensor configuration.

## Conclusions

In the article, we have presented the evaluation methodology that can be used to test and compare different SLAM algorithms based on data from LiDARs, IMU and odometry. We also describe a general simulation testing approach together with appropriate tools and discuss the advantages and disadvantages of such an evaluation strategy.

In our case, we have used a simulated environment introduced and verified in^12^ to compare different hardware configurations of Google Cartographer SLAM algorithm to be deployed in an actual robot used for decontamination (thus the possible placement of different sensors was limited). We examined eight different hardware configurations based on 3 LiDARs 2D, one LiDAR 3D, IMU and wheel odometry in two experimental environments - a laboratory room and a hallway. We have compared these configuration in terms of localization accuracy (measured based on SLAM errors: Relative Pose Error and Absolute Trajectory Error) and mapping performance (based on generated maps analysis regarding the occurrence of key objects representations in occupancy grids and general accuracy of generated maps, i.e. shape of the examined room etc.).

The results of our analysis have shown that in our case the hardware configuration consisting of three LiDARs 2D, IMU and wheel odometry seems to be the best choice in terms of localization accuracy, mapping performance and cost-efficiency. Also, our results show that adding data from additional sensors, i.e. IMU and odometry, can improve the stability of the SLAM algorithm even without using the loop closure mechanism. Due to the placement of the LiDARs at the bottom of the robot (it was the most suitable place regarding the robot’s shape), some walls of the room have not been mapped properly, as they were simply obscured by tall equipment. Nevertheless, the overall mapping accuracy was very good and sufficient for the application. The results of our tests conducted for larger areas or long hallways show that LiDAR sensor range is also important. In the case of such environments it might be better to use sensors with larger ranges (e.g. our combination of 3D LiDAR and IMU sensors provided the best accuracy in the tested environment). On the other hand, in our case, 3D LiDARs had to be placed on the top of the robot, therefore some of the lower objects in the laboratory room could not be detected, which can decrease the applicability of the robot in the indoor environments (e.g. the robot can drive into such an object). This shows that 3D LiDARs would be better for lower robots. These results show that an optimal hardware configuration should not only take into account the environment, but also the robot’s construction, purpose and capabilities. Nevertheless, using all of the examined configurations, we obtained relatively good performance. It seems that Google Cartographer using all of the examined data sources and configurations can be used in the indoor environments similar to the examined ones.

Simulation-based testing allows performing more robust tests and more tests in general (as it can be easily automated), it provides access to precise ground truth, perfectly identical testing conditions in real time and can be used to model larger environments than ones we have access to. Generally, it is a cost-effective alternative to real-world evaluation in the early stages of product design and to reduce the number of necessary real-life tests. It is important to mention that a simulation is never as detailed as the real world, therefore the real-world verification is recommended in the final stages of the development process—before the real-world deployment of any solution. Nevertheless, simulation testing can greatly reduce the time and cost needed to select the right sensor configuration.

## Supplementary Information

Supplementary Information.

## Data Availability

The datasets generated and analysed during this study are included in this published article [and its supplementary information files].
