# Supplementary figures and images for: Finding the best hardware configuration for 2D SLAM in indoor environments via simulation based on Google Cartographer
Source: Sci Rep. 2022 Nov 5;12:18815. doi: 10.1038/s41598-022-22938-y (PMC9637188; doi:10.1038/s41598-022-22938-y)

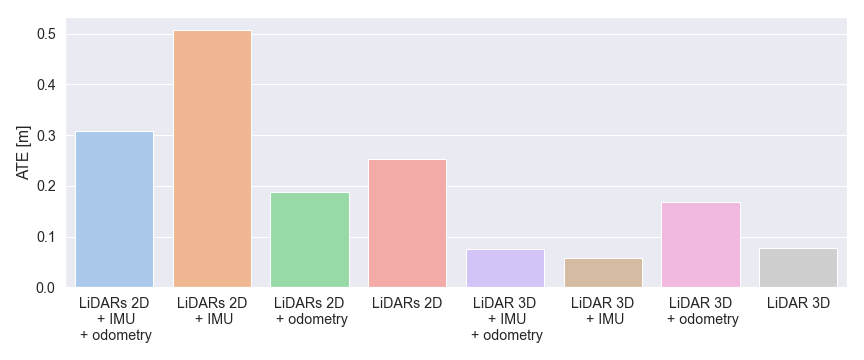

Supplement: Supplementary file 1 — Supplementary Information. [file 41598_2022_22938_MOESM1_ESM.zip › SLAM_readings/tunel/tunel_ate_max.png]

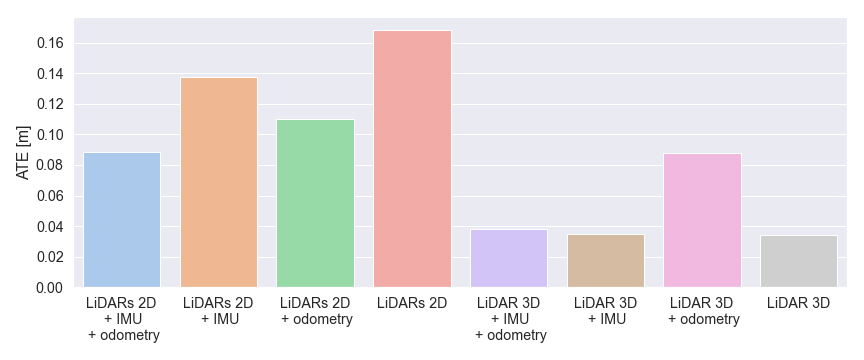

Supplement: Supplementary file 1 — Supplementary Information. [file 41598_2022_22938_MOESM1_ESM.zip › SLAM_readings/tunel/tunel_ate_mean.png]

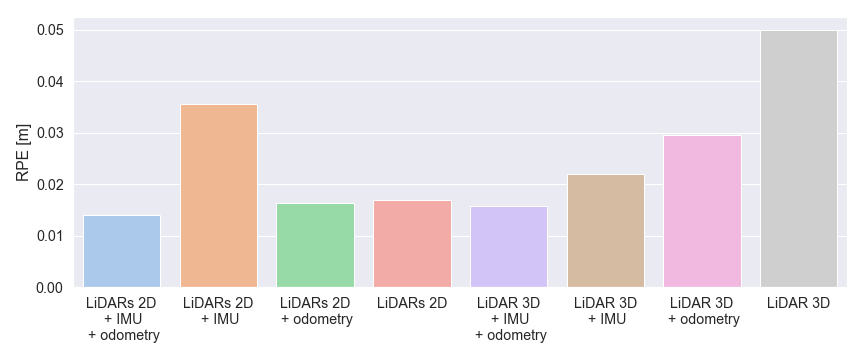

Supplement: Supplementary file 1 — Supplementary Information. [file 41598_2022_22938_MOESM1_ESM.zip › SLAM_readings/tunel/tunel_rpe_max.png]

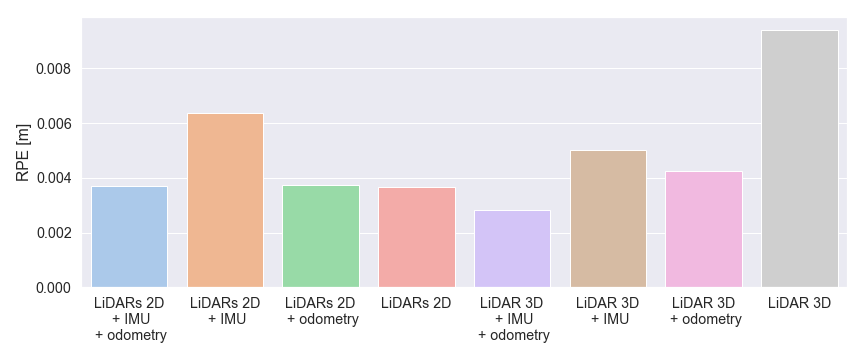

Supplement: Supplementary file 1 — Supplementary Information. [file 41598_2022_22938_MOESM1_ESM.zip › SLAM_readings/tunel/tunel_rpe_mean.png]

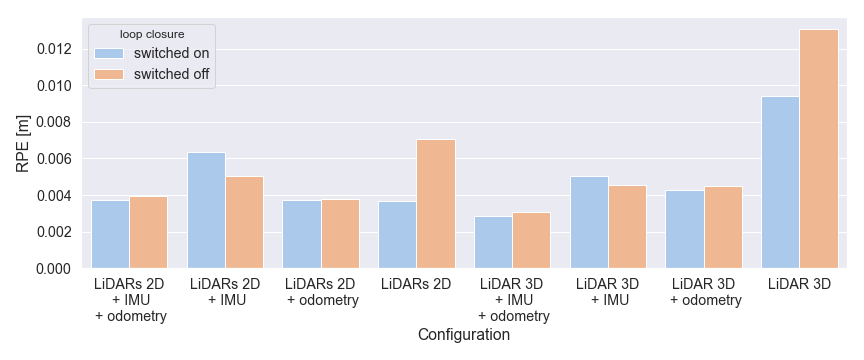

Supplement: Supplementary file 1 — Supplementary Information. [file 41598_2022_22938_MOESM1_ESM.zip › SLAM_readings/tunel_no_loop_closure/tunel_rpe_mean_comparison.png]
